# Carnitine Conjugate of Nipecotic Acid: A New Example of Dual Prodrug

**DOI:** 10.3390/molecules14093268

**Published:** 2009-08-31

**Authors:** Carmela Napolitano, Martina Scaglianti, Emanuela Scalambra, Stefano Manfredini, Luca Ferraro, Sarah Beggiato, Silvia Vertuani

**Affiliations:** 1 Department of Pharmaceutical Sciences, University of Ferrara, Via Fossato di Mortara 17-19, I-44100 Ferrara, Italy; 2 Department of Clinical and Experimental Medicine, Section of Pharmacology, University of Ferrara, Via Fossato di Mortara 17-19, I-44100 Ferrara, Italy; 3 Ambrosialab srl, Via Saragat 4a, I-44100 Ferrara, Italy

**Keywords:** L-carnitine, molecular conjugation, increased brain delivery, prodrug, nipecotic acid

## Abstract

As a novel example of improved entry of poorly delivered drugs into the brain by means of nutrient conjugates, L-carnitine was conjugated to nipecotic acid and the capacity to antagonize PTZ-induced convulsions of this novel entity was evaluated.

## Introduction

Distribution of drugs to the central nervous system (CNS) is one of the major problems of current therapy of brain diseases. Difficulties in the crossing of the brain blood barrier (BBB) often impair the efficacy of valuable drugs. In the past, most of the attempts to overcome this drawback have been directed to the amelioration of the lipophilic properties through the preparation of prodrugs by formation of reversible linkages with suitable groups [1,2]. A new approach that takes into account the recent progress in molecular cloning and the expression of transporters genes is being applied too. It is now clear that membrane transporters of native compounds also take part in drug transport in various tissues. Several specific transporters have been identified in the brain capillary endothelia, and among them some of those involved in the active supply of nutrients (i.e., glucose, amino acids) have been used to prepare prodrugs with improved CNS penetration [3]. We have already reported a significative example of the latter approach [4] and here describe a new application of this prodrug concept. 

We have investigated the conjugation of L-carnitine (L-C), a vital cofactor for the mitochondrial oxidation of fatty acids [5] that posses specific carrier-mediated transport systems for its uptake [6,7], with nipecotic acid as a tool to demonstrate the capability of L-C to behave as a drug carrier in the brain. The choice of L-C as *carrier* introduces a novel concept referred to as a “double prodrug” approach, which involves the preparation of a novel single molecular entity with therapeutic effects resulting from two diverse, but synergistic mechanisms of action. A suitable covalent attachment of carnitine and nipecotic acid may have a significant therapeutic value: the crossing of the BBB of the conjugate and the controlled release of both drugs *in vivo* through enzymatic hydrolysis could result not only in a good bioavailability of anticonvulsivant agent in the brain, but also in the prevention of induced carnitine deficiency disorders. 

## Results and Discussion

**Scheme 1:**
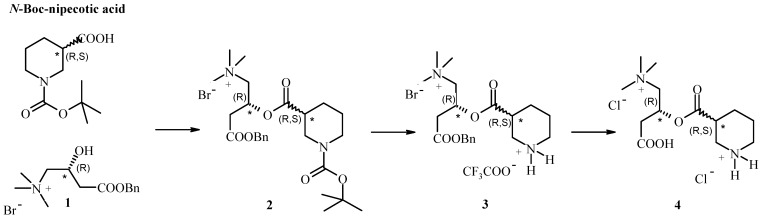
Conjugation of L-C to nipecotic acid.

The strategy for the conjugation of L-C to nipecotic acid is described in Scheme 1. Nipecotic acid and L-C needed previous protection at the amino and carboxylic functions respectively. Thus nipecotic acid was protected as its *tert*-butoxycarbonyl derivative and L-C as the benzyl ester **1** under standard conditions. Several attempts were made in order to improve the reactivity of the carboxylic function of *N*-Boc-nipecotic acid and thus access to conjugate **2**. The most significant improvements in the efficiency of the reaction were obtained converting the carboxylic acid into an acyl chloride employing oxalyl chloride in the presence of a catalytic amount of a mixture of DMAP/TEA. The ester **2** was finally deprotected by treatment with trifluoroacetic acid, followed by classical hydrogenolysis, the molecule was purified by ion exchange solid phase extraction to afford final compound **4** in good yield.

To support the possibility that L-C may work as a shuttle for drugs, and thus that conjugation with L-C could be a means to improve the entry into the brain of drugs that do not easily penetrate the BBB, we evaluated the effects of the systemic injection of nipecotic acid (racemic mixture), L-C and conjugate **4** on pentylenetetrazole (PTZ)-induced convulsions in mice [8]. In the saline group, the injection of PTZ (80 mg/kg) induced tonic convulsions with a latency of 677 ± 51 s. As shown in Table 1, the ip injection of **4 **(0.75 mmol/kg) significantly increased the latency of the appearance of PTZ-induced tonic convulsions, while, as expected [9,10], the administration of nipecotic acid or L-C, at the same dose (0.75 mmol/kg), were ineffective. No lethality was observed in any of the groups. 

**Table 1:** Effect of ip injection of nipecotic acid (0.75 mmol/kg), L-C (0.75 mmol/Kg) or conjugate **4** (0.75 mmol/kg) on pentylenetetrazole (PTZ)-induced convulsions in mice.

Treatment	Latency to convulsions (s)
Control (saline + PTZ)	677 ± 51
Nipecotic acid + PTZ	645 ± 50
L-carnitine + PTZ	664 ± 62
**4** + PTZ	1021 ± 55^a^

^a^*P* < 0.05, significantly different from control and nipecotic acid groups, according to ANOVA followed by the Newman–Keuls test for multiple comparisons.

**Figure 1:**
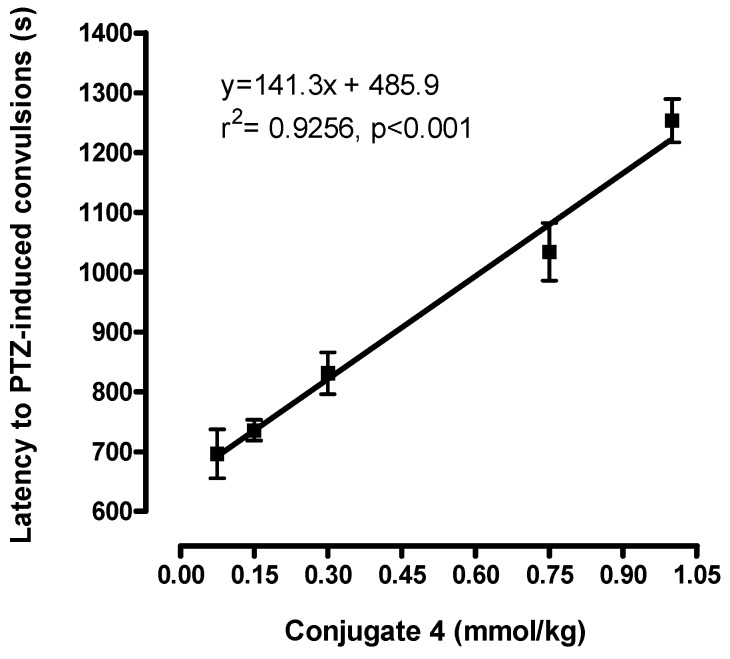
Dose-response effect of conjugate **4** (0.75 mmol/kg ip) on pentylenetetrazole (PTZ)-induced convulsions in mice. Each point represents the mean ± SEM of 6-7 animals.

Interestingly, as shown in Figure 1, the effect of conjugate **4** on PTZ-induced convulsions in mice was dose-dependent in the range of 0.075-1 mmol/kg.

To prove that compound **4** can cross the BBB, nipecotic acid concentration in mouse brains was evaluated. The mice were treated with saline solution, nipecotic acid (0.75 mmol/kg) or compound **4 **(0.75 mmol/kg)**,** respectively. Thirty minutes after injection the mice were sacrificed and nipecotic acid concentration in brain was determined by HPLC analysis. The saline treated group did not show any traces of nipecotic acid in the brain, nor did the nipecotic treated group, whereas, the mice treated with compound **4** showed 600 nmol/g concentration of nipecotic acid in the brain (Table 2). 

**Table 2:** Nipecotic acid concentration in mice brain, 30 minute after injection of saline, nipecotic acid (0.75 mmol/kg) or compound **4** (0.75 mmol/kg).

Treatment	Nipecotic acid (nmol/g)^a^
Control (saline)	n.d.
Nipecotic acid	n.d.
Compound 4	600

^a ^Limit of quantification (LOQ) of nipecotic acid was ~58 nmol/g.

## Conclusions

The conjugation of L-C to nipecotic acid is a demonstration that the approach of delivery of drugs by means of nutrients using their endogenous transporters is of great potential value. This result further confirms our previous discovery of a similar role exerted by ascorbic acid and opens another window on this interesting tool. Further studies are currently on going in order to confirm and extend this possibility to other active molecules endowed with poor or no BBB crossing properties.

## Experimental Section

### General

All moisture sensitive reactions were performed under an argon atmosphere. Reaction courses and product mixtures were routinely monitored by thin-layer chromatography (TLC) on silica gel precoated F254 Merck plates with detection under 254 nm UV lamp and/or by spraying with a diluted potassium permanganate solution. Column chromatography was performed with Merck 60-200 mesh silica gel. Compound **4 **was obtained by purification with Supelco DSC-SAX, ion exchange (capacity 0.14 meq/g). Melting points were determined with a capillary apparatus and are uncorrected. ^1^H-NMR spectra were recorded on a Bruker Advance 400 spectrometer. All drying operations were performed over anhydrous magnesium or sodium sulphate. Nipecotic acid was protected as its *tert*-butoxycarbonyl (Boc) derivative as reported in [11].

*Synthesis of l-carnitine benzyl ester* (**1**): Benzyl bromide (440 mL, 3.71 mmol) was added to a solution of l-carnitine (500 mg, 3.1 mmol) in a 1.6:1 mixture dioxane/ DMF (25 mL). The solution was heated to 120 °C for 4 h. After cooling to rt, volatiles were evaporated under vacuum. Purification by chromatography over silica gel (DCM/ MeOH = 95:5) of the crude residue afforded 560 mg of the pure **1 **(72%). White solid; m.p. 180-183 °C. ^1^H-NMR (CD_3_OD) δ 2.62-2.66 (m, 2H), 3.22 (s, 9H), 3.46 (dd, *J* = 4.6 Hz, *J* = 6.0 Hz, 2H), 4.56-4.67 (m, 1H), 5.15 (d, *J* = 12.4 Hz, 1H), 5.19 (d, *J* = 12.4 Hz, 1H), 7.28-7.42 (m, 5H). 

*[3-Benzyloxycarbonyl-2(R)-(1-tert-butyloxycarbonyl-piperidine-3(R,S)-carbonyloxy)propyl]-trimethyl-ammonium bromide* (**2**): To a stirred solution of *N*-Boc nipecotic acid (1.146 g, 5.0 mmol) in dry THF (5.0 mL) were added oxalyl chloride (525 μL) and dry DMF (10 μL). The resulting yellow pale solution was stirred at room temperature for 2 h. Volatiles were evaporated under vacuum and the residue was dissolved in dry THF (10 mL). A portion of this solution (400 μL) was added dropwise at 0 °C to a solution of **1** (50.0 mg, 0.20 mmol), DMAP (35.0 mg, 0.29 mmol) and TEA (0.82 mL) in dry THF (2.7 mL). The resulting mixture was stirred at room temperature for 18 h. Volatiles were evaporated under vacuum and the residue purified by chromatography over silica gel (DCM/ MeOH = 8:2) to afford 82 mg of the pure conjugate **2** (89%). Colorless oil; ^1^H-NMR (CD_3_OD) δ 1.36-1.49 (m, 10H), 1.52-1.74 (m, 2H), 1.84-2.01 (m, 1H), 2.38-2.53 (m, 1H), 2.83-2.94 (m, 2H), 3.06-3.20 (m, 11H), 3.61-3.80 (m, 2H), 3.83-3.97 (m, 2H), 5.15 (s, 2H), 5.60-5.71 (m, 1H), 7.31-7.42 (m, 5H). 

*[3-Benzyloxycarbonyl-2(R)-(piperidine-3(R,S)-carbonyloxy)-propyl]-trimethyl-ammonium bromide trifluoroacetate* (**3**): TFA (1.8 ml, 23.7 mmol) was added dropwise to a solution of **2 **(591 mg, 1.08 mmol) in dry CH_2_Cl_2_. (2.6 mL). The mixture was stirred at room temperature for 3 h. Volatiles were removed under vacuum to afford **3 **as yellow pale oil (quantitative). ^1^H-NMR (CD_3_OD) δ 1.57-2.18 (m, 5H), 2.84-3.33 (m, 16 H), 3.46 (bt, *J* = 13.2 Hz, 1H), 3.74 (dd, *J* = 2.4 Hz, *J* = 14.0 Hz, 1H), 3.88-3.98 (m, 1H), 5.14 (d, *J* =12.4 Hz, 1H), 5.18 (d, *J* =12.4 Hz, 1H), 5.65-5.73 (m, 1H), 7.29-7.42 (m, 5H).

*[3-Carboxy-2(R)-(piperidine-3(R,S)-carbonyloxy)-propyl]-trimethyl-ammonium dichloride* (**4**): 10% Pd/C (200 mg) was added to a solution of **3 **(703 mg, 1.33 mmol) in MeOH (20 mL) and the mixture was stirred 5 h under hydrogen atmosphere. The mixture was filtered on a pad of Celite and the solvent removed under vacuum to afford 550 mg of trifluoroacetic-bromide salt that was purified by cromatogaphy over silica-bound quaternary amine a strong anion exchange (eluant MeOH = 100) to get 407mg of the pure dichloride salt **4** (93%). ^1^H-NMR (DMSO) δ 1.55-1.77 (m, 3H), 1.93-2.05 (m, 1H), 2.72-2.93 (m, 5H), 3.11-3.33 (m, 11H), 3.64-3.68 (m, 1H), 3.85-3.99 (m, 1H), 5.46-5.47 (m, 1H), δ 9.0-9.5 (m, 2H), δ 12.5-12.9 (broad s, 1H),

### Effects of the drugs on pentylenetetrazol-induced seizures

Swiss albino male mice (25-30 g body weight) were used. In any experimental session a maximum of 15 animals were acutely injected i.p. with saline (control), nipecotic acid (0.75 mmol/kg), L-C (0.75 mmol/Kg) or compound **4 **(0.075, 0.15, 0.3, 0.75 and 1 mmol/Kg). Twenty five minutes after the treatment, all mice were subcutaneously injected with pentylenetetrazole (80 mg/kg) and the animals were observed for the following 30 min by an investigator who was unaware of the treatment. The latency (in sec) to appearance of generalized tonic convulsions and lethality were measured to evaluate the effects of the treatments on pentylenetetrazole-induced convulsions. Lethality was defined as the percentage of the animal died within 60 min after pentylenetetrazole injection.

### Determination of nipecotic acid in mouse brains

*Nipecotic acid brain standard curve*. Mice were anesthetized and sacrificed by decapitation, the brains were rapidly removed, rinsed with saline solution and homogenized in ice-cold 0.1 N HCl. The resultant solutions were spiked with different concentrations of aqueous nipecotic acid solution and the homogenates were centrifuged. The supernatants were collected filtered and then derivatized with phenylisothiocyanate (PITC) to give a phenylthiocarbamyl derivative of nipecotic acid (PTC-nipecotic acid). The filtrates were frozen, evaporated to dryness and dissolved in coupling buffer (100 μL, acetonitrile-pyridine-triethylamine-water, 10:5:2:3) under nitrogen, frozen again and evaporate to dryness. The samples were dissolved in coupling buffer (100 μL) of to which was added PITC (5 μL) and after 5 minutes at room temperature the solutions were frozen and evaporated to dryness. Three mouse brains were used for each concentration of nipecotic acid on the standard curve and triplicate aliquots of each brain sample were assayed by HPLC as described below. The brain standard curve was constructed as described in literature [12] by plotting the mean AUC against the amount of nipecotic acid. The curve was derived over a range of 20-250 pmol of nipecotic acid, the minimum sensitivity being around ~10 pmol of nipecotic acid or ~58 nmol of nipecotic acid/g brain tissue [12]. Mouse brains to which no nipecotic acid was added were subjected to these same procedures and served as controls [12]. 

*HPLC-separation of PTC-Nipecotic Acid*. The reverse-phase separation was performed by following the method described by Nassereddine-Sebaei *et al*. [12]. Briefly, a Waters Novo-pak C18 column (4 µm particle-size, 15 cm in length and 3.9 mm in diameter), maintained at 52 °C, was used. The mobile phase consisted of 90% solvent A (0.05 M ammonium acetate solution adjusted to pH 6.8 with phosphoric acid) and 10% of solvent B (10% 1.0 ammonium acetate solution adjusted to pH 6.8 with phosphoric acid, 10% methanol, 44% acetonitrile, 36% water); the flow rate was 1.0 mL/min and the samples were analyzed a wavelength of 284 nm by using an UV/Vis variable wavelength detector. 

*Distribution of nipecotic acid in mouse brain*. Mice were treated with saline, nipecotic acid (0.75 mmol/kg) or compound **4 **(0.75 mmol/kg), they were anesthetized and sacrificed by decapitation 30 min after injection. The brains were processed as previously described and the filtrates were derivatized as described above. The amount of nipecotic acid in brain was calculated with the brain standard curve, from the average AUC values for three triplicates for each brain supernatant. 

### Statistical evaluation

Statistical analysis was performed by ANOVA followed by the Newman-Keuls test for multiple comparisons.
